# PCA‐based respiratory motion modeling for individualized PTV margin optimization in NSCLC radiotherapy

**DOI:** 10.1002/acm2.70652

**Published:** 2026-06-07

**Authors:** Huy Dang Quang, Cong Nguyen Thanh, Trang Hoang Thi Kieu, Tao Chau Van

**Affiliations:** ^1^ Department of Radiotherapy, Institute of Oncology and Nuclear Medicine Military Hospital 175 Ho Chi Minh City Vietnam; ^2^ Department of Nuclear Physics, Faculty of Physics and Engineering Physics University of Sciences Ho Chi Minh City Vietnam; ^3^ Vietnam National University Ho Chi Minh City Vietnam

**Keywords:** individualized PTV margin, non‐small cell lung cancer (NSCLC), principal component analysis (PCA), respiratory motion management

## Abstract

**Background:**

Respiratory motion remains a major challenge in radiotherapy for non‐small cell lung cancer (NSCLC), often requiring a balance between target coverage and normal tissue sparing.

**Introduction:**

This study aimed to develop and validate a clinically implementable, individualized PTV margin strategy by integrating principal component analysis (PCA) with key clinical predictors.

**Methods:**

A cohort of 61 NSCLC patients (Stage III) underwent 4DCT simulation. 4DCT datasets were used both for motion modeling and as a reference planning approach (ITV‐based). The proposed method was compared against conventional 3DCT and 4DCT‐based plans. PCA was applied to the deformable vector fields to extract dominant motion modes and quantify directional amplitudes (*A_mean_*). Clinical correlations with tumor motion were assessed. An individualized margin recipe, $\;M_{d}=\sqrt{{\left(2.5\;\Sigma \right)}^{2}+{\left(0.7\sigma \right)}^{2}+{\left(\alpha *F_{pos}*F_{T}*A_{mean}\right)}^{2}}$ was implemented, incorporating a Location Factor $F_{pos}$ and a T stage Factor ($F_{T}$) derived from cohort‐specific data. Dosimetric plans (3DCT, Individualized‐3DCT and 4DCT) were compared using QUANTEC constraints.

**Results:**

Tumor location (*p* = 0.007) and T stage (*p* = 0.04) were identified as significant predictors of S–I motion amplitude. Middle/lower lobe tumors and early T1–T2 stages exhibited higher mobility (≥ 15 mm). By applying adaptive weighting factors ($F_{pos}$= 1.2 for lower lobes; $F_{T}$= 0.9 for T3–T4), the individualized 3D plans achieved a significant reduction in PTV volume compared to 3D plans (410.0 ± 170.0 cm^3^ vs. 460.2 ± 179.1 cm^3^, *p* = 0.002). The individualized margin approach reduced lung dose compared with conventional 3DCT planning (V20: 30.0% vs. 40.5%), while remaining comparable to the 4DCT‐based reference plan (28.9%). Other OARs remained within tolerance limits.

**Conclusions:**

Integrating PCA‐derived motion with clinical weighting provides a practical framework for individualized margin design, improving lung sparing while maintaining target coverage.

## 1 INTRODUCTION

Respiratory‐induced tumor motion is a well‐recognized challenge in thoracic radiotherapy and represents a major contributor to geometric uncertainty during treatment delivery.^1^, ^2^, ^3^, ^4^ Lung tumors can exhibit substantial displacement during normal breathing, particularly in the superior–inferior direction, potentially compromising target coverage or increasing irradiation of surrounding normal tissues.^5^, ^6^, ^7^To compensate for this motion, planning target volume (PTV) margins are routinely applied during treatment planning. However, the selection of margin size and geometry remains a delicate balance between geometric robustness and normal tissue sparing, with direct implications for lung toxicity and treatment tolerance.^8^, ^9^

Four‐dimensional computed tomography (4DCT) has become the clinical reference standard for assessing respiratory motion in lung cancer radiotherapy.^2^, ^3^, ^10^By reconstructing images across multiple phases of the breathing cycle, 4DCT enables direct visualization and quantification of tumor displacement throughout the respiratory cycle. In this study, tumor displacement was further quantified from DVF‐based voxel‐wise motion within the GTV relative to the reference phase. Directional components were obtained by projection onto SI, AP, and LR axes in three dimensions. Despite this capability, margin definition in routine clinical practice often continues to rely on population‐based isotropic expansions derived from empirical experience or guideline recommendations.^8^, ^11^, ^12^This apparent discrepancy reflects the practical reality that detailed motion information is not always fully translated into individualized margin strategies.

Several clinical studies have demonstrated that tumor motion is highly patient‐specific and strongly influenced by tumor location.^5^, ^6^, ^13^Superior–inferior displacement typically dominates respiratory motion, particularly for tumors located in the lower lobes, whereas anterior–posterior and left–right components are generally smaller but remain clinically relevant.^7^, ^14^Uniform isotropic margins therefore risk overexpansion in some directions while under‐representing motion in others, potentially leading to unnecessary increases in lung dose without proportional gains in target coverage.^9^, ^15^Such dose escalation is clinically important, given the established association between lung dose–volume parameters and the risk of radiation pneumonitis.^9^, ^16^, ^17^

Advanced motion‐management strategies, including respiratory gating, tumor tracking, breath‐hold techniques, and adaptive radiotherapy, have been developed to mitigate the effects of respiratory motion.^4^, ^18^While these techniques can substantially reduce motion‐related uncertainty, their implementation require specialized hardware, additional imaging, increased staff expertise, and longer treatment times. As a result, they may not be routinely available in all centers, particularly in resource‐limited settings or high‐throughput clinical environments.^18^, ^19^, ^20^Consequently, many institutions continue to rely on conventional 3DCT‐based planning with population‐based margins, despite recognizing the limitations of this approach.

In this context, there is growing interest in pragmatic strategies that leverage existing 4DCT data to improve margin personalization without introducing substantial operational burden. Physics‐informed respiratory motion modeling techniques, including those based on principal component analysis (PCA), have been investigated as compact representations of patient‐specific motion derived from 4DCT.^19^, ^21^PCA enables complex motion trajectories to be described using a small number of dominant components, offering a potential pathway toward individualized margin definition. PCA‐based motion modeling has been previously investigated for respiratory motion characterization (Li et al,^22^Zhang et al.^21^), and margin recipes such as the van Herk formulation are widely established in radiotherapy. However, the clinical integration of these approaches into a practical workflow for individualized PTV margin selection remains limited. The present study aims to bridge this gap by combining motion modeling, clinical factors, and margin formulation into a unified, clinically applicable framework.

However, most prior studies have focused on technical validation or motion reconstruction accuracy rather than on clinically relevant endpoints such as lung dose, toxicity risk, and workflow feasibility.^23^, ^24^, ^25^

The present study evaluates the clinical feasibility of translating a physics‐informed PCA‐based Singular Value Decomposition model into individualized PTV margins for patients with locally advanced NSCLC. Target volume definitions were based on the International Commission on Radiation Units and Measurements (ICRU) Reports 50 and 62, which establish the conceptual framework for GTV, CTV, and PTV. In this study, these principles were extended by incorporating motion‐informed anisotropic margins derived from patient‐specific respiratory motion.^26^

## 2 MATERIALS AND METHODS

### 2.1 Patient population

The cohort included 61 consecutive patients with stage III NSCLC treated with definitive radiotherapy. Inclusion criteria required high‐quality free‐breathing 4DCT datasets and curative treatment intent. Patients with prior thoracic radiotherapy or significant breathing irregularities were excluded to ensure the reliability of respiratory phase sorting.^10^, ^27^

**FIGURE 1:**
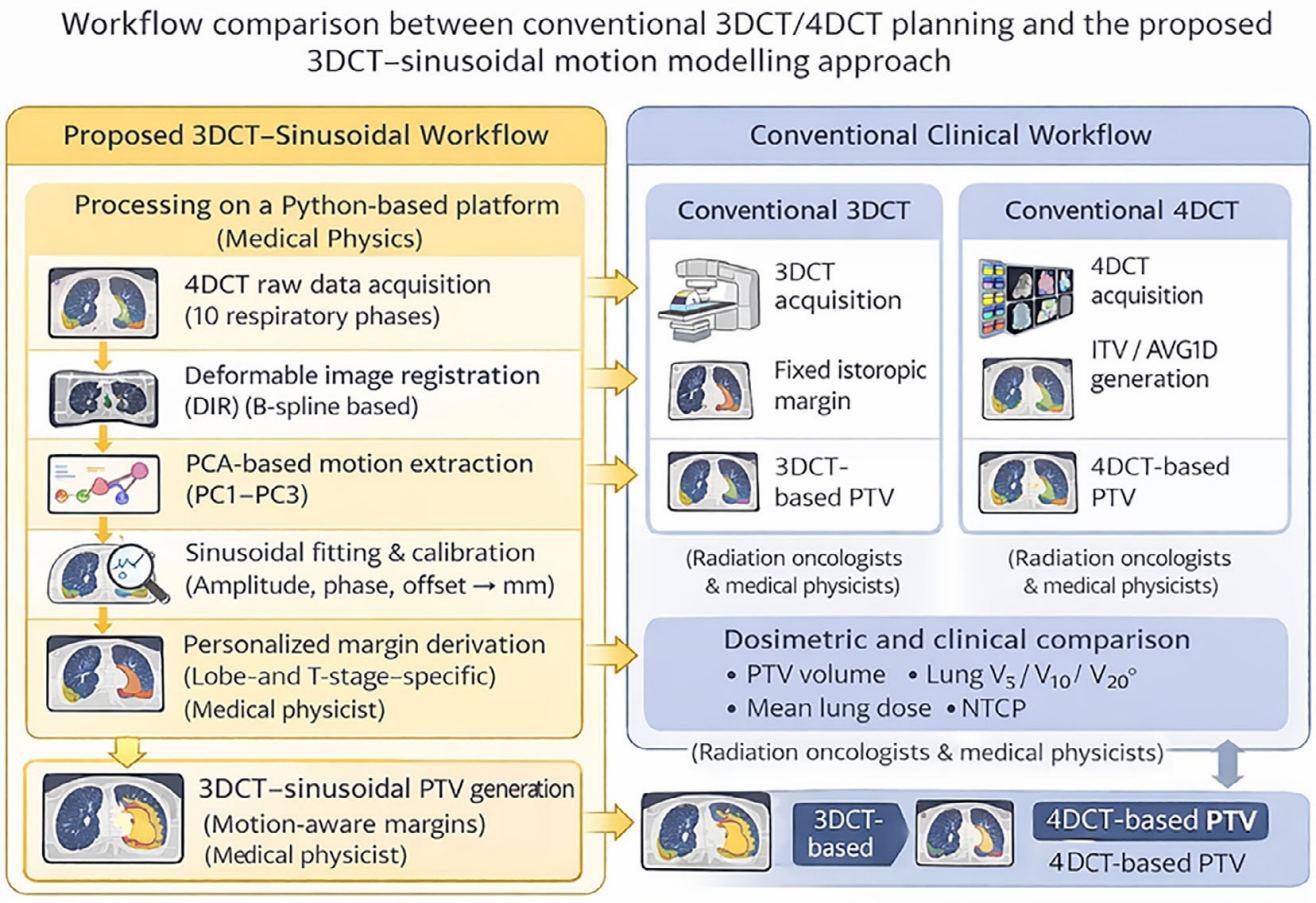
Clinical workflow for motion‐informed individualized margin generation.

The overall workflow of the proposed method is illustrated in Figure 1. The approach consists of four main steps: (1) Deformable registration of 4DCT phases, (2) PCA‐based motion extraction, (3) Sinusoidal modeling, and (4) Derivation of individualized PTV margins. These steps enable integration of motion information into a 3DCT‐based planning framework.

**FIGURE 2:**
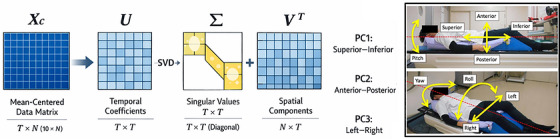
Singular value decomposition (SVD) formulation of the PCA‐based respiratory motion model.

**FIGURE 3:**
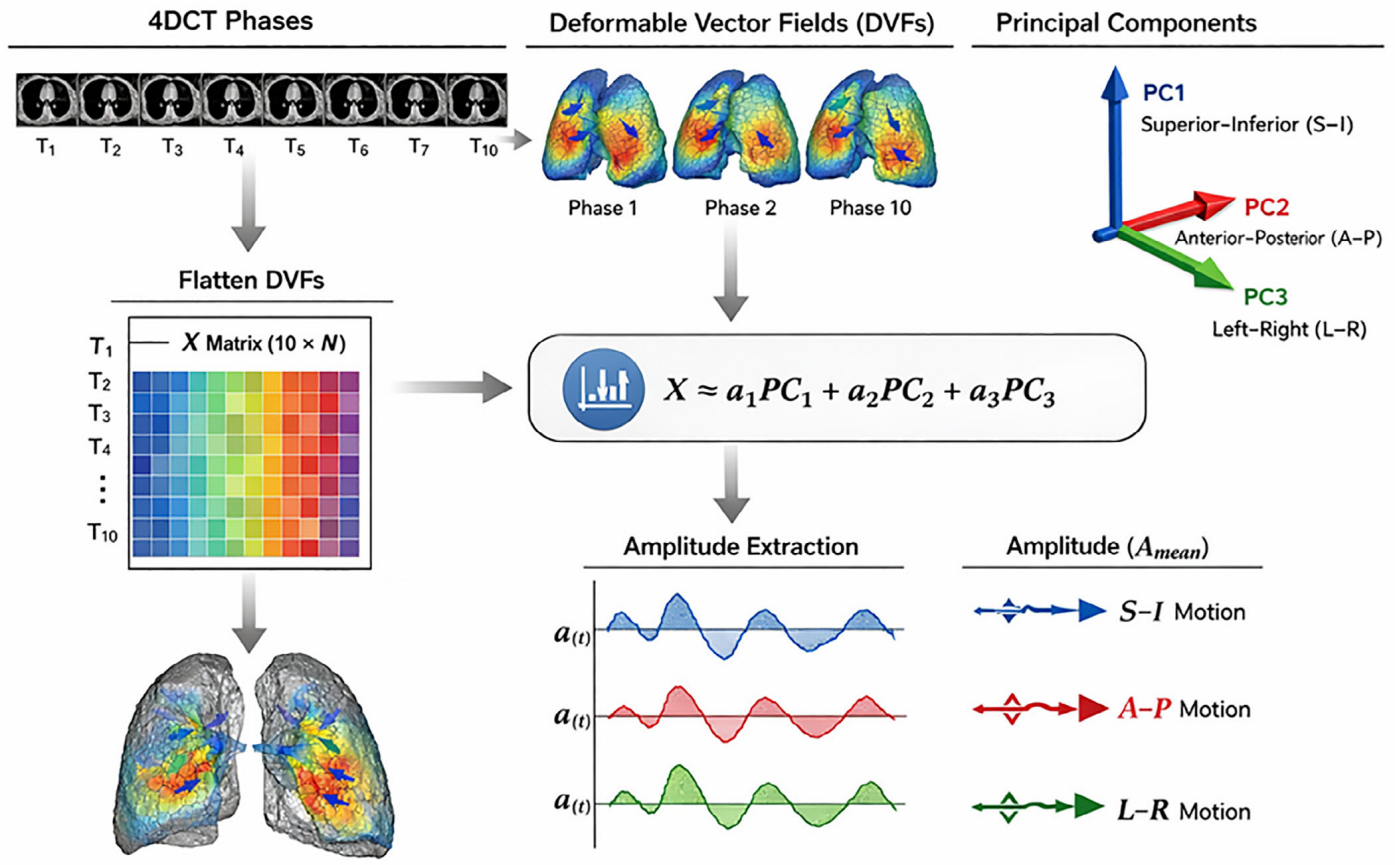
Workflow of PCA‐based respiratory motion modeling from 4DCT‐derived deformable vector fields (DVFs).

### 2.2 4DCT acquisition and motion quantification

Free‐breathing 4DCT simulation was performed using standard clinical protocols, with ten respiratory phases reconstructed via amplitude‐based sorting.^2^, ^3^ Gross tumor volumes were delineated on a reference phase and propagated across all respiratory phases using deformable image registration, as described in previous clinical implementations.^27^, ^28^

All imaging data were processed within the Monaco (version 5.11, Elekta) treatment planning system. Both 3DCT and respiratory‐correlated 4DCT datasets (10 phases, T00–T90) were used for target delineation and comparative planning. For the 3DCT‐based plan, the gross tumor volume (GTV) was delineated on the free‐breathing CT dataset. The clinical target volume (CTV) was generated by applying a 5–8 mm expansion to the GTV, in accordance with ESTRO recommendations. PTV margins were then applied anisotropically to account for setup and motion uncertainties, using 10 mm in the anterior–posterior (AP) and left–right (LR) directions and 15 mm in the superior–inferior (SI) direction.

For the 4DCT‐based plan, the internal target volume (ITV) was defined as the union of GTV contours across all respiratory phases (T00–T90), or derived from maximum intensity projection (MIP) images. A uniform setup margin of 5 mm was then applied to the ITV to generate the corresponding PTV. Treatment planning was performed on the average intensity projection (AIP) dataset. Deformable image registration was applied to propagate GTV contours from the reference phase to all respiratory phases.

For the individualized margin approach, patient‐specific motion amplitudes derived from PCA‐based analysis of DVF fields were incorporated into margin estimation. Directional motion components were projected onto SI, AP, and LR axes, and combined with setup uncertainties to generate individualized PTV margins.

All target delineations were reviewed by two independent radiation oncologists with senior and junior clinical experience levels, respectively, to ensure contouring consistency and quality. All treatment plans (3DCT‐based, individualized margin, and 4DCT‐based) were generated using identical beam configurations and optimization objectives. The only variable modified between plans was the PTV definition, allowing for direct assessment of the dosimetric impact of margin adaptation.

### 2.3 Physics‐informed PCA–SVD motion modeling

To construct a compact and clinically interpretable representation of respiratory‐induced tumor motion, PCA was applied to deformable vector fields (DVFs) derived from 4DCT images.

#### 2.3.1 Data representation and dimensionality reduction

Instead of operating on high‐dimensional image intensities (512×512×77 voxels per phase), PCA was performed on motion fields. For each respiratory phase (t), the 3D DVF was reshaped into a one‐dimensional vector:

(1) $$x_{t}\in R^{N}$$

Where N represents the total number of voxel‐wise displacement components (including S–I, A–P, and L–R directions). Stacking all phases yielded a data matrix:

(2) $$X=\left[\begin{array}{c} x_{1}\\ x_{2}\\ \begin{array}{c} x_{3}\\ \text{…}\\ x_{T} \end{array} \end{array}\right]\in R^{N\times T},T=10$$

Importantly, PCA was performed across the temporal dimension (phases), ensuring that the number of samples remained small (*T* = 10), thereby avoiding the curse of dimensionality. A reference phase was selected for deformable image registration and contour propagation. In this study, the end‐expiration phase (T50) was used as the reference phase, as it is generally associated with more stable and reproducible tumor positioning. All displacement fields were computed relative to this reference phase, and GTV contours were propagated from the reference phase to all other respiratory phases.

#### 2.3.2 SVD formulation (Figure 2)

PCA was computed using singular value decomposition (SVD):

(3) $$x_{c}=U\Sigma V^{T}$$

Where:

- $X_{c}:$ Mean‐centered data matrix
- $U\in \;R^{T\;\times \;T}$: Contains orthogonal spatial basis functions (principal motion modes)
- Σ: Diagonal matrix of singular values representing motion energy
- $V\in \;R^{N\;\times \;T}$: Encodes the temporal evolution of motion.

The mean‐centered motion matrix $x_{t}\in \;R^{N\;\times T}$ where T represents the number of respiratory phases and N the number of spatial degrees of freedom, was decomposed using singular value decomposition as shown in Equation (3). In this formulation, the columns of V represent the spatial principal components (motion modes), while the product UΣ contains the corresponding temporal coefficients describing the evolution of each mode across respiratory phases. This decomposition enables a low‐rank representation of respiratory motion, where dominant motion patterns are captured by a small number of principal components. The first three principal components were interpreted as dominant respiratory motion modes rather than predefined anatomical directions. Because PCA identifies data‐driven modes of maximal variance, each component may contain mixed SI, AP, and LR displacement contributions. Therefore, directional motion amplitudes were quantified by projecting the reconstructed DVF‐derived displacement vectors onto the anatomical SI, AP, and LR axes, rather than assuming a one‐to‐one correspondence between PC order and anatomical direction. For each retained principal component, the relative directional contribution was calculated as the fraction of displacement energy along each anatomical axis:

(4) $$C_{k,d}=\frac{\sum_{v}{\left(PC_{k,d}\left(v\right)\right)}^{2}}{\sum_{d\in \left\{SI,AP,LR\right\}}\sum_{v}{\left(PC_{k,d}\left(v\right)\right)}^{2}}\;$$

Where $C_{k,d}$ represents the contribution of anatomical direction d to principal component k, and v denotes voxel position. This formulation transforms high‐dimensional voxel‐wise motion into a compact, physically interpretable model suitable for clinical margin estimation.

#### 2.3.3 Motion reconstruction (Figure 3)

Respiratory motion was approximated using a reduced‐order model:

(5) $$X_{c}\approx \;a_{1}\left(t\right)\;PC_{1}+a_{2}\left(t\right)\;PC_{2}+a_{3}\left(t\right)\;PC_{3}$$

Where the first three principal components **(PC1–PC3)** captured > 90% of total motion variance. From a physical perspective: As shown in Figure S2.1, the first three PC1–PC3 were interpreted as dominant data‐driven motion modes. Direction‐specific amplitudes were obtained by projecting reconstructed displacement fields onto the SI, AP, and LR anatomical axes. where $a_{i}\left(t\right)$ represents the temporal coefficient of each motion mode. For each patient, the centered motion vector at respiratory phase (*t*), denoted by $X_{c,t}$, was projected onto the retained principal components to obtain the temporal coefficients:

(6) $$a_{i}\left(t\right)={\;X}_{c,t}^{T}\;PC_{i}$$

Where *i = 1, 2, 3*. Equivalently, under the SVD formulation $X_{c}=\;U\;\Sigma \;V^{T}$, the temporal coefficients correspond to the entries of UΣ.

#### 2.3.4 Amplitude extraction and clinical interpretation

The temporal coefficients $a_{i}\left(t\right)$ describe the respiratory trajectory along each motion mode. The corresponding motion amplitude was defined as:

(7) $$A_{i}^{\mathrm{mm}}=\frac{\mathrm{ma}x_{t}\;a_{i}\;\left(t\right)\;-\;\mathrm{mi}n_{t}\;a_{i}\;\left(t\right)}{2}\;$$

PCA decomposes the displacement field into spatial modes and corresponding coefficients, which are dimensionless by definition. Therefore, the PCA coefficients themselves do not directly represent physical displacement. To obtain physically meaningful motion amplitudes, the displacement fields were reconstructed from the retained components and projected onto anatomical axes, from which amplitudes were computed in millimeters. These values were subsequently used as motion inputs in the individualized margin formulation.

Temporal coefficients are subsequently used to derive motion amplitudes ($A_{mean}$), enabling direct clinical interpretation and integration into individualized margin design. All data processing and analysis were performed using Python (version 3.11). The implementation utilized standard scientific libraries, including NumPy, SciPy, scikit‐learn, and pandas for data handling, PCA computation, and statistical analysis. Curve fitting was performed using *scipy.optimize*, and statistical metrics were computed using *scikit‐learn*. The repository includes the complete computational workflow for motion modeling, including preprocessing, deformable registration, PCA/SVD analysis, and margin calculation. An archived version of the repository with a permanent DOI is available at: https://doi.org/10.5281/zenodo.19397400. Although formal quantitative validation was not performed, all propagated contours were visually reviewed by experienced clinicians to ensure anatomical consistency across respiratory phases.

### 2.4 Individualized margin recipe (Indiv‐3D)

Patient‐specific directional margins were derived from the modeled motion amplitudes in each axis. These individualized margins were compared with conventional population‐based isotropic margins commonly applied in routine clinical practice.^8^, ^15^Margin concepts were consistent with established geometric uncertainty frameworks and international reporting recommendations.^11^, ^12^

To ensure clinically adequate target coverage, setup uncertainty was incorporated using the widely adopted van Herk margin formulation:^8^

(8) β=2.5Σ+0.7σ

Where Σ and σ represent systematic and random setup errors, respectively. In the proposed framework, respiratory motion amplitude derived from DVF‐based analysis was incorporated as an additional uncertainty component. Because setup‐related errors and respiratory motion arise from different physical processes—namely patient positioning and intra‐fraction anatomical motion—they were assumed to be statistically independent. Under this assumption, the combined margin was calculated using a root‐sum‐square (quadrature) approach, which is commonly applied when independent uncertainty sources are combined. This approach avoids overestimation that may occur with linear summation and provides a physically consistent estimate of the overall geometric uncertainty. Therefore, the individualized margin formulation integrates setup and motion‐related uncertainties in a unified framework while preserving consistency with established margin theory. This formulation aims to ensure that 90% of patients receive at least 95% of the prescribed dose to the clinical target volume (CTV), and is therefore considered a robust population‐based margin standard in radiotherapy.

In the present study, patient‐specific respiratory motion was explicitly integrated into margin design. Directional motion amplitudes derived from 4DCT/PCA modeling were converted into effective systematic excursions using a scaling factor (*α* = 0.5), reflecting the transition from peak‐to‐peak motion to mean positional deviation. The resulting margin formulation combines population‐based setup uncertainty with individualized motion information, enabling a more patient‐specific yet clinically acceptable margin definition.

Unlike conventional isotropic expansions, individualized margins $M_{d}$ were derived using a quadratic combination to account for independent error contributions:

(9) $$M_{d}=\sqrt{{\left(2.5\;\Sigma \right)}^{2}+{\left(0.7\sigma \right)}^{2}+{\left(\alpha \;*\;F_{pos}\;*\;F_{T}\;*\;A_{mean}\right)}^{2}}\;$$

Patient‐specific directional margins were first estimated independently along the SI, AP, and LR axes based on DVF‐derived motion amplitudes and setup uncertainties. For applications requiring a single isotropic margin, these directional components were subsequently combined into a scalar value using a root‐sum‐square formulation, as expressed in Equation (10), where $A_{\mathrm{mean}}$ represents the aggregated motion amplitude. Directional margins ($M_{SI},M_{AP},M_{LR}$) were computed independently. When a single margin value was required for comparison or implementation, these were combined into an effective isotropic margin using Equation (10).

$A_{\mathrm{mean}}$: Patient‐specific directional motion amplitude derived from the PCA model. **Clinical Significance**: This factor reflects the positive correlation between the tumor's proximity to the diaphragm and its respiratory motion amplitude.

$F_{\mathrm{pos}}$: The Tumor Location Factor is a geometric correction parameter integrated into the PTV margin formula. It is designed to adaptively adjust the safety margin width based on the precise anatomical coordinates of the tumor within the thorax

Established Values:

- $F_{pos}$ = 1.2 (Middle/Lower Lobe): Applied to tumors with a high risk of mobility due to their location near the hemi‐diaphragm—the region exhibiting the largest motion amplitude (mean: 17.1 ± 5.10 mm). In our cohort, 36.8% of middle/lower lobe tumors demonstrated significant superior–inferior (S–I) displacement (≥ 15 mm) (*p* = 0.007).
- $F_{pos}$ = 1.0 (Upper Lobe): Applied to tumors with greater anatomical stability. This allows for a more constrained PTV volume, thereby maximizing the sparing of healthy lung parenchyma.

$F_{T}$: *Option 1* ($F_{T}$ = 1.1): Applied to T1–T2 tumors. These smaller, non‐invasive lesions demonstrate higher mobility and “free‐floating” characteristics, requiring a slightly expanded margin to prevent target under‐dosage. *Option 2* ($F_{T}$: = 0.9): Applied to T3–T4 tumors. Due to their larger volume and frequent tethering to adjacent structures (e.g., mediastinum or chest wall), these tumors exhibit restricted motion. This reduction factor minimizes unnecessary irradiation of healthy lung tissue (V_20_ and MLD).

α: A weighting coefficient (set at 0.5) to ensure target coverage while minimizing normal tissue irradiation.

This formulation reflects Gaussian error propagation and avoids potential overestimation associated with linear summation. It provides a more conservative and theoretically grounded estimate of total geometric uncertainty, particularly in patients with large respiratory motion amplitudes. Together, these formulations aim to balance robustness and normal tissue sparing, while maintaining compatibility with established clinical margin design principles.

The weighting factors $F_{pos}$ and $F_{T}$ were empirically derived from our institutional data analysis. Specifically, $F_{pos}$ was categorized based on the significant correlation between tumor location and S‐I motion amplitude (*p* = 0.007), where lower‐lobe lesions exhibited a 40% probability of large‐scale displacement (≥ 15 mm). Similarly, $F_{T}$ was determined by the T stage dependency of motion (*p* = 0.04), accounting for the restricted mobility observed in advanced T3–T4 tumors compared to the high‐mobility profiles of T1–T2 lesions. To assess the robustness of the tumor location factor ($F_{\mathrm{pos}}$) and T‐stage factor ($F_{T}$), internal validation was performed using bootstrap resampling (1000 iterations). In each iteration, the dataset was resampled with replacement, and the weighting factors were recalculated to evaluate their variability and stability. The distribution of the estimated parameters was summarized using mean values and 95% confidence intervals. The clinical decision‐making process for lung cancer patients was standardized using a comprehensive workflow (see Methods S3). This protocol begins with 3DCT simulation and a detailed clinical assessment, including tumor location, T stage, and respiratory patterns.

### 2.5 Dosimetric and NTCP evaluation

For each patient, three planning strategies were compared: Isotropic 3DCT (Iso‐3D), Individualized 3D (Indiv‐3D), and full 4DCT‐based planning. Dosimetric endpoints included PTV volume, lung $V_{20}$, and mean lung dose (MLD), which are well‐established predictors of radiation pneumonitis.^9^, ^16^, ^17^Estimated normal tissue complication probability (NTCP) for radiation pneumonitis was calculated based on established dose–volume relationships:^9^, ^16^, ^17^NTCP was calculated using the Lyman–Kutcher–Burman (LKB) model with parameters derived from QUANTEC guidelines. Doses to other organs at risk, including mean heart dose, esophageal dose metrics, and maximum spinal cord dose, were also recorded in accordance with clinical tolerance guidelines.^12^, ^29^ The analysis focused on relative differences within each patient rather than absolute plan optimization.

(10) $$\mathrm{NTCP}=f\;\left(V_{20},\;\mathrm{MLD}\right)$$

All treatment plans (3DCT‐based, individualized 3D [Indiv‐3D], and 4DCT‐based) were generated using identical beam configurations, dose prescriptions, and optimization objectives. The only variable modified between plans was the PTV definition. This ensured that any observed dosimetric differences were attributable solely to margin adaptation rather than differences in planning strategy.

### 2.6 Statistical analysis

Statistical analysis was performed using SPSS v26.0 and Python code.

- **Descriptive Statistics**: Continuous variables were reported as mean ± standard deviation (SD)
- **Comparative Analysis**: A paired t‐test or Wilcoxon signed‐rank test (depending on normality) was used to compare PTV volumes, dosimetric parameters, and NTCP (**Python code**) values between the three planning strategies.
- **Subgroup Analysis**: Patients were stratified by tumor location (upper vs. lower lobe) and motion amplitude to evaluate the selective benefit of margin personalization.
- **Significance**: A p‐value of < 0.05 was considered statistically significant.

Overall comparisons among planning strategies were performed using repeated‐measures ANOVA.

#### 2.6.1 Sensitivity analysis

A sensitivity analysis was performed to evaluate the robustness of the proposed margin formulation with respect to key parameters, including setup uncertainties (Σ, σ) and the motion scaling factor (*α*). Systematic (*Σ*) and random (*σ*) setup uncertainties were assumed to be **2.5 mm** and **1.5 mm**, respectively, based on institutional setup error analysis and consistent with previously reported values in lung radiotherapy,^8^while *α* was 0.5 to reflect different assumptions in converting peak‐to‐peak motion into effective displacement.

Across all tested scenarios, the individualized margin approach consistently resulted in reduced PTV volumes compared with isotropic margins, while maintaining stable lung dose metrics. Variations in Σ and σ primarily affected the absolute magnitude of margins but did not alter the relative dosimetric advantage of the proposed method.

## 3 RESULTS

The study cohort (*N* = 61) exhibited significant inter‐patient variability in respiratory‐induced tumor motion. Analysis of the 4DCT datasets revealed that displacement was most pronounced in the superior–inferior (S–I) direction. Direction‐specific amplitudes were quantified by projecting the displacement vectors onto the anatomical SI, AP, and LR axes. The mean motion amplitudes were 7.5 ± 4.5 mm in the SI direction, 4.2 ± 2.7 mm in the AP direction, and 3.6 ± 1.7 mm in the LR direction. This directional asymmetry was particularly evident in lower‐lobe tumors (Table 1). In the revised analysis, all motion amplitudes were derived directly from the DVF‐based displacement fields, which are expressed in physical units (millimeters).

**TABLE 1:** Directional tumor motion amplitudes derived from PCA‐based analysis.

Direction	Mean Motion Amplitude (mm)
Superior–Inferior (S–I)	7.5 ± 4.5
Anterior–Posterior (A–P)	4.2 ± 2.7
Left–Right (L–R)	3.6 ± 1.7

Directional contribution analysis showed that the leading respiratory motion mode was predominantly associated with SI displacement, whereas the remaining modes contained mixed AP and LR components. Therefore, anatomical direction–specific amplitudes were reported after projection of the reconstructed displacement fields onto SI, AP, and LR axes. These values served as the motion inputs for the individualized margin formulation rather than the final margin values themselves.

**FIGURE 4:**
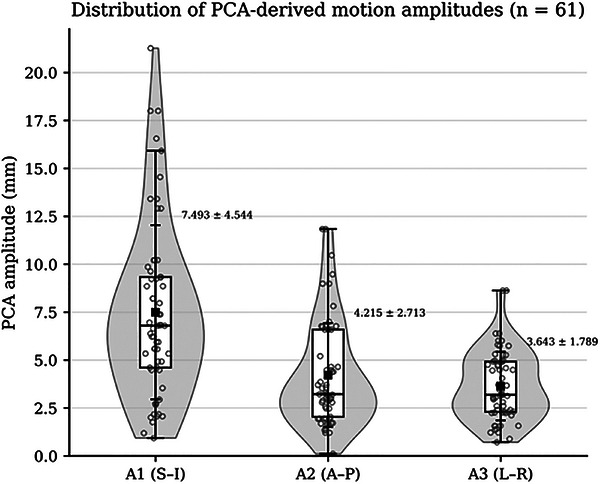
Distribution of PCA‐derived motion amplitudes across the cohort (*n* = 61). The amplitudes A_1_, A_2_, and A_3_ correspond to the peak‐to‐peak variation of the temporal coefficients associated with the first three principal motion components (PC1–PC3), representing the superior–inferior, anterior–posterior, and left–right directions, respectively.

Figure 4 shows a raincloud‐style visualization combining violin density, boxplot summary, and individual patient data points for the first three principal motion components. Patient‐specific amplitudes were computed as half the peak‐to‐peak variation across respiratory phases. PC1 exhibited the largest motion magnitude, followed by PC2 and PC3.

Baseline patient characteristics and respiratory‐induced tumor motion amplitudes derived from 4DCT. Motion is reported as mean ± standard deviation in the superior–inferior (S–I), anterior–posterior (A–P), and left–right (L–R) directions.

**FIGURE 5:**
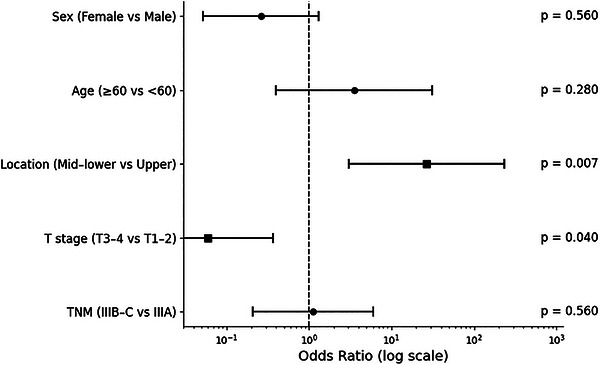
Forest plot illustrating the association between clinical factors and large superior–inferior (S–I) tumor motion (≥15 mm).

Tables 2, 3, 4 summarize the association between clinical factors and tumor motion across all anatomical directions. Tumor location and T stage demonstrated statistically significant associations with respiratory motion amplitude. Middle–lower lobe tumors exhibited significantly larger motion amplitudes than upper lobe tumors (14.6 ± 6.8 mm vs. 5.2 ± 2.7 mm, *p* = 0.007). Similarly, T1–T2 tumors demonstrated greater motion amplitudes compared with T3–T4 tumors (13.8 ± 7.4 mm vs. 7.5 ± 4.1 mm, *p* = 0.040). In particular, middle/lower‐lobe tumors exhibited a substantially higher proportion of large motion compared with upper‐lobe tumors. A trend toward greater motion amplitude was observed in the T1–T2 group compared to T3–T4; however, this finding should be interpreted with caution due to the limited sample size (*n* = 8) in the early‐stage subgroup. Logistic regression analysis (Figure 5) demonstrated that tumor location was significantly associated with large S–I motion (OR > 1, *p* = 0.007), indicating increased motion in middle–lower lobe tumors. T stage was also significantly associated (*p* = 0.040), with advanced‐stage tumors showing reduced motion. No significant associations were observed for sex, age, or TNM stage, as confidence intervals crossed unity. SI motion ≥ 15 mm was considered clinically relevant high respiratory mobility. Based on these findings, tumor location and T stage were incorporated into the margin formulation as weighting factors $F_{\mathrm{pos}}$ and $F_{T}$, respectively. *Percentages represent proportions within each subgroup (row‐wise), indicating the distribution of SI motion (<* *15 mm* vs. *≥15 mm) for each clinical category. Bootstrap analysis demonstrated that both* $F_{\mathrm{pos}}$ *and* $F_{T}$ *remained stable across resampled datasets, with narrow confidence intervals. The dosimetric impact of the individualized margin strategy was consistent across bootstrap samples, indicating stability of the proposed framework. Patients were stratified into three motion risk categories based on the Directional Margin Calculation (M_d_): Low (M_SI_ < 5 mm), Intermediate (5–10 mm), and High (> 10 mm). As illustrated in* *Methods* *S3* *, the imaging technique was then tailored to the risk level, utilizing 3DCT, Individualized‐3DCT (PCA‐sinusoidal), or 4DCT (ITV‐based) respectively*.

**TABLE 2:** Association between clinical factors and superior–Inferior (S–I) tumor motion.

Clinical factor	Subgroup	*N*	<15 mm n (%)	≥15 mm n (%)	Mean ± SD (mm)	*p*‐value
Gender	Male	18	14 (77.8)	4 (22.2)	5.5 ± 4.2	0.56
	Female	43	40 (93.0)	3 (7.0)	7.1 ± 4.3	
Age	< 60	21	20 (95.2)	1 (4.8)	6.2 ± 3.8	0.28
	≥ 60	40	34 (85.0)	6 (15.0)	9.4 ± 5.9	
Tumor location	Upper lobe	41	40 (97.6)	1 (2.4)	5.2 ± 2.7^*^	0.007
	Middle–lower lobe	20	12 (60.0)	8 (40.0)	14.6 ± 6.8^*^	
T stage	T1–T2	8	4 (50.0)	4 (50.0)	13.8 ± 7.4^†^	0.040
	T3–T4	53	50 (94.3)	3 (5.7)	7.5 ± 4.1^†^	
TNM stage	IIIA	12	10 (83.3)	2 (16.7)	8.2 ± 4.9	0.56
	IIIB–IIIC	49	40 (81.6)	9 (18.4)	9.0 ± 5.5	

* Statistically significant difference between upper lobe and middle–lower lobe tumors (*p* < 0.01).

^†^ Statistically significant difference between T1–T2 and T3–T4 tumors (*p* < 0.05).

**TABLE 3:** Association between clinical factors and Anterior–Posterior (A–P) tumor motion.

Clinical factor	Subgroup	*n*	<10 mm n (%)	≥10 mm n (%)	Mean ± SD (mm)	*p*‐value
Gender	Male	18	16 (88.9)	2 (11.1)	4.5 ± 2.9	0.29
	Female	43	40 (93.0)	3 (7.0)	4.1 ± 2.6	
Age	< 60	21	20 (95.2)	1 (4.8)	4.1 ± 2.5	0.87
	≥ 60	40	38 (95.0)	2 (5.0)	4.3 ± 2.8	
Tumor location	Upper lobe	42	40 (95.2)	2 (4.8)	3.9 ± 2.4	0.32
	Middle–lower lobe	19	17 (89.5)	2 (10.5)	4.8 ± 3.2	
T stage	T1–T2	10	8 (80.0)	2 (20.0)	4.3 ± 2.9	0.90
	T3–T4	51	50 (98.0)	1 (2.0)	4.2 ± 2.6	
TNM stage	IIIA	14	12 (85.7)	2 (14.3)	4.2 ± 2.7	0.99
	IIIB–IIIC	47	45 (95.7)	2 (4.3)	4.2 ± 2.7	

**TABLE 4:** Association between clinical factors and Left–Right (L–R) tumor motion.

Clinical Factor	Subgroup	*n*	<10 mm n (%)	≥10 mm n (%)	Mean ± SD (mm)	*p*‐value
Gender	Male	18	16 (88.9)	2 (11.1)	3.7 ± 1.8	0.98
	Female	43	40 (93.0)	3 (7.0)	3.6 ± 1.7	
Age	< 60	21	20 (95.2)	1 (4.8)	3.5 ± 1.6	0.95
	≥ 60	40	38 (95.0)	2 (5.0)	3.6 ± 1.8	
Tumor location	Upper lobe	42	40 (95.2)	2 (4.8)	3.4 ± 1.5	0.32
	Middle–lower lobe	19	17 (89.5)	2 (10.5)	4.1 ± 2.1	
T stage	T1–T2	10	8 (80.0)	2 (20.0)	3.7 ± 1.9	0.99
	T3–T4	51	50 (98.0)	1 (2.0)	3.6 ± 1.6	
TNM stage	IIIA	14	12 (85.7)	2 (14.3)	3.6 ± 1.7	0.92
	IIIB–IIIC	47	40 (85.1)	7 (14.9)	3.7 ± 1.8	

### 3.1 Respiratory motion and model‐derived margins

The cohort exhibited substantial inter‐patient variability in motion. Using the physics‐informed PCA model, these motion components were translated into individualized margins ($M_{d}$). These values represented a significant reduction compared to the standard 10–15 mm isotropic margins used in the Iso‐3D plans. The accuracy and agreement of the PCA–sinusoidal motion model were further evaluated using RMSE, coefficient of determination (R^2^), and Bland–Altman analyses (Section S1). The model demonstrated high accuracy, with sub‐millimetric RMSE observed across the majority of analyzed slices (Figure S1.1). Strong agreement between model‐predicted and measured motion trajectories was also confirmed by Bland–Altman analysis, which showed minimal systematic bias and narrow limits of agreement (Section S1, Figures S1.1–S1.2).

Substantial inter‐patient variability in tumor motion was observed across the cohort. Mean displacement was greatest in the superior–inferior direction, with lower‐lobe tumors exhibiting larger motion amplitudes compared with upper‐lobe lesions.

### 3.2 Dosimetric and toxicity impact

**FIGURE 6:**
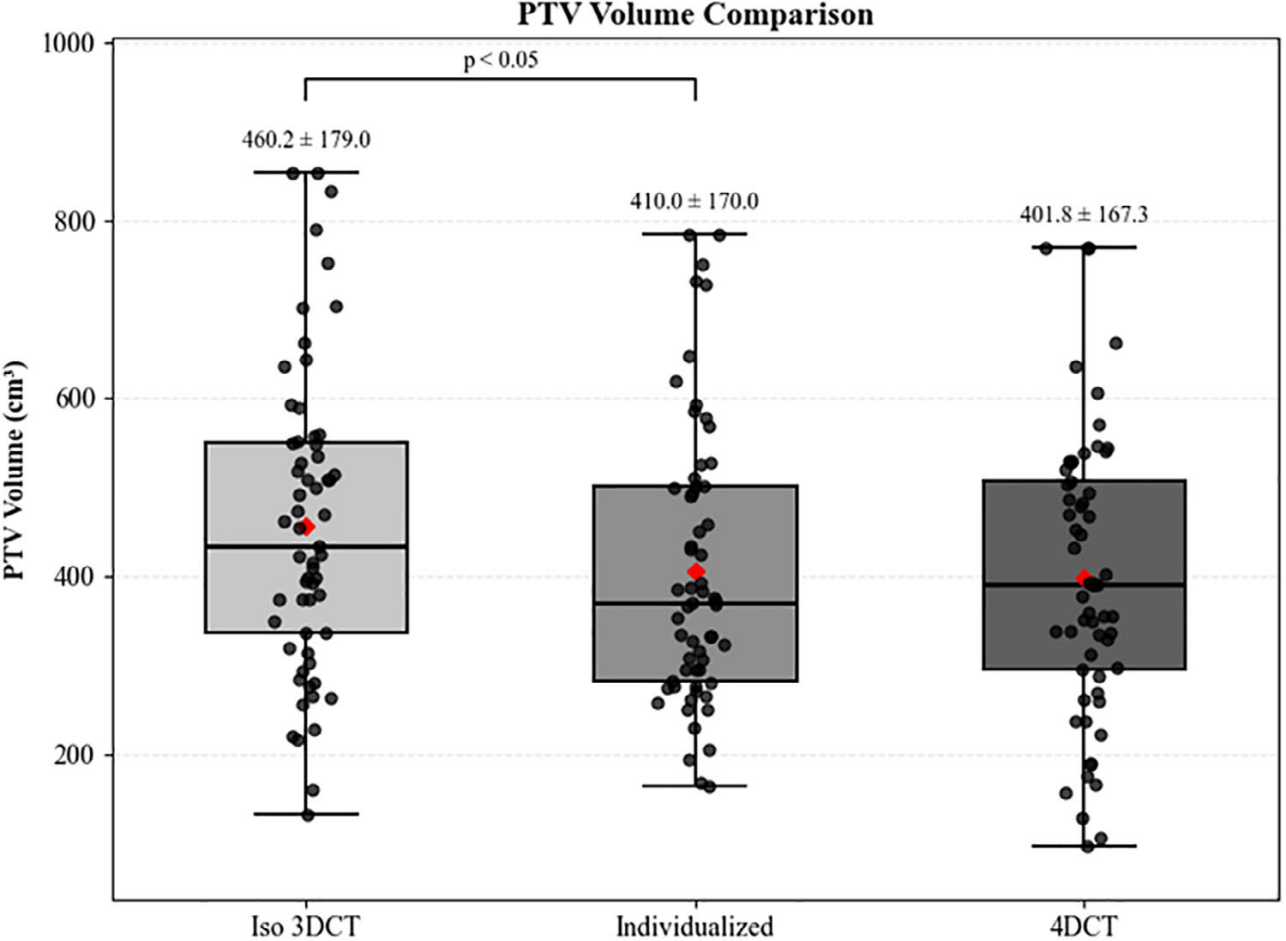
Boxplot comparison of PTV across three radiotherapy strategies.

Figure 6 provides a comprehensive comparison of isotropic, individualized, and 4DCT‐based PTV margin strategies, demonstrating consistent dosimetric advantages associated with motion‐adaptive approaches. Across all evaluated scenarios, both individualized margins (Indiv‐3D) and 4DCT‐based planning resulted in a significant reduction in PTV volume compared with conventional isotropic margins (Iso‐3D) (all *p* < 0.05). This reduction translated directly into improved normal tissue sparing, as evidenced by significantly lower lung V20 (%) and mean lung dose (MLD) values in both motion‐informed strategies relative to Iso‐3D.

### 3.3 Impact on lung 20%, mean lung dose, and estimated NTCP

The magnitude of reduction varied by tumor location. Beyond geometric effects, individualized margin strategies resulted in clinically meaningful reductions in lung dose metrics. Both lung V20 and mean lung dose were reduced compared with conventional isotropic 3DCT‐based planning, consistent with established dose–toxicity relationships.^9^, ^16^ ^17^

**FIGURE 7:**
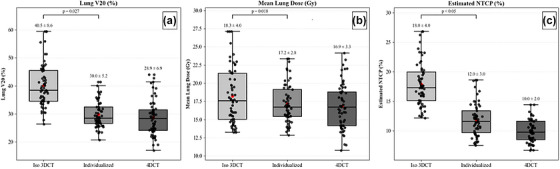
Comparative dosimetric and radiobiological outcomes: (A) Lung V20, (B) Mean Lung Dose, and (C) NTCP.

These dosimetric improvements translated into a corresponding decrease in estimated NTCP for radiation pneumonitis (from approximately 18%–22%–10%–14%, *p* < 0.05; Figure 7), with values approaching those obtained using 4DCT‐based planning. Importantly, lung sparing was not achieved at the expense of increased dose to other organs at risk. No statistically significant differences were observed in mean cardiac dose, oesophageal dose metrics, or maximum spinal cord dose across planning strategies, and all spinal cord doses remained well below accepted clinical tolerance limits^12^, ^29^ (Table S1). A quantitative comparison of target volume, lung dose metrics, estimated NTCP, and selected organ‐at‐risk doses between planning strategies is provided in Table 5. Importantly, no statistically significant differences were detected between Indiv‐3D and 4DCT‐based approaches for any of the evaluated endpoints, including PTV volume, lung V20, and MLD.

**TABLE 5:** Summary of key PTVs and dosimetric outcomes (*N* = 61).

Parameter	Isotropic 3DCT (Baseline)	Individualized‐ 3DCT (Proposed)	4DCT Planning (Reference)	*p*‐value (Iso vs. Indiv/Indiv vs. 4DCT)
PTV volume (cm^3^)	460.2 ± 179.1	410.0 ± 170.0	401.8 ± 167.3	0.002 / 0.256
Lung V_20_ (%)	40.5 ± 8.6	30.0 ± 5.2	28.9 ± 6.9	0.027 / 0.156
Mean lung dose (Gy)	18.3 ± 4.0	17.2 ± 2.8	16.9 ± 3.3	0.018 / 0.350
Estimated NTCP (%)	18.0 ± 4.0	12.0 ± 3.0	10.0 ± 2.0	0.003 / 0.475

The observed consistency of these findings across variations in superior–inferior motion amplitude and tumor location further supports the robustness of the proposed framework, although external validation is required to confirm generalizability of the individualized margin framework.

Subgroup analysis demonstrating the impact of individualized margins according to tumor motion characteristics. Patients with larger superior–inferior tumor motion amplitudes (or lower‐lobe tumors) exhibited greater reductions in PTV and lung dose when individualized margins were applied.

Despite a significant reduction in PTV volume, target coverage was preserved across all planning strategies. The individualized margin approach demonstrated comparable CTV and GTV coverage metrics (D95 and V95) relative to the 4DCT‐based reference plan, with no statistically significant differences (*p* > 0.05) (Table 6).

**TABLE 6:** Comparison of target coverage metrics across planning strategies.

Metric	3DCT (Iso)	Indiv‐3D (PCA)	4DCT (Ref)	*p*‐value
PTV D95 (%)	97.2 ± 1.1	96.9 ± 1.3	97.4 ± 1.0	0.28
PTV V95 (%)	99.1 ± 0.9	98.9 ± 1.0	99.0 ± 0.8	0.35
CTV D95 (%)	98.1 ± 1.2	97.8 ± 1.4	98.3 ± 1.1	0.32
CTV V95 (%)	99.2 ± 0.8	99.0 ± 1.0	99.3 ± 0.7	0.41
GTV D95 (%)	99.0 ± 0.9	98.7 ± 1.1	99.2 ± 0.8	0.28
GTV V95 (%)	99.6 ± 0.5	99.4 ± 0.6	99.7 ± 0.4	0.36
Minimum Dose (Gy)	58.2 ± 2.5	57.8 ± 2.7	58.5 ± 2.3	0.30

Furthermore, phase‐aware dose evaluation indicated that accumulated target coverage remained within clinically acceptable limits. The only variable modified between plans was the PTV definition. Therefore, the observed reductions in lung dose metrics can be attributed directly to the reduction in PTV volume rather than differences in planning technique.

The difference in target coverage between individualized and 4DCT‐based plans was minimal (ΔD95 < 1%). Metrics such as accumulated CTV and GTV D95 were estimated to assess the impact of respiratory motion on target coverage. Results demonstrate that motion‐inclusive dose evaluation remains within clinically acceptable limits, supporting the robustness of the individualized margin strategy (Table 7).

**TABLE 7:** Accumulated target dose evaluation for individualized and 4DCT‐based plans.

Metric	Indiv‐3D	4DCT
Accumulated CTV D95 (%)	97.5 ± 1.60	98.1 ± 1.30
Accumulated GTV D95 (%)	98.3 ± 1.20	98.9 ± 1.00

	Indiv‐3D vs. 4DCT	
ΔD95 (%)	−0.6 ± 0.8	

**FIGURE 8:**
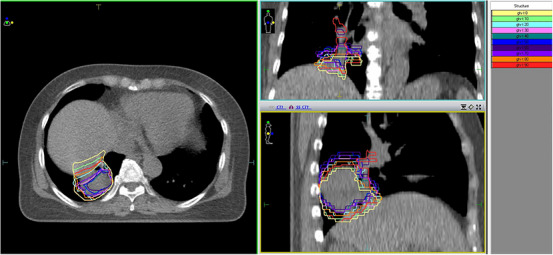
Representative patient illustrating respiratory‐induced tumor motion across 10 phases of 4DCT (T00–T90).

**FIGURE 9:**
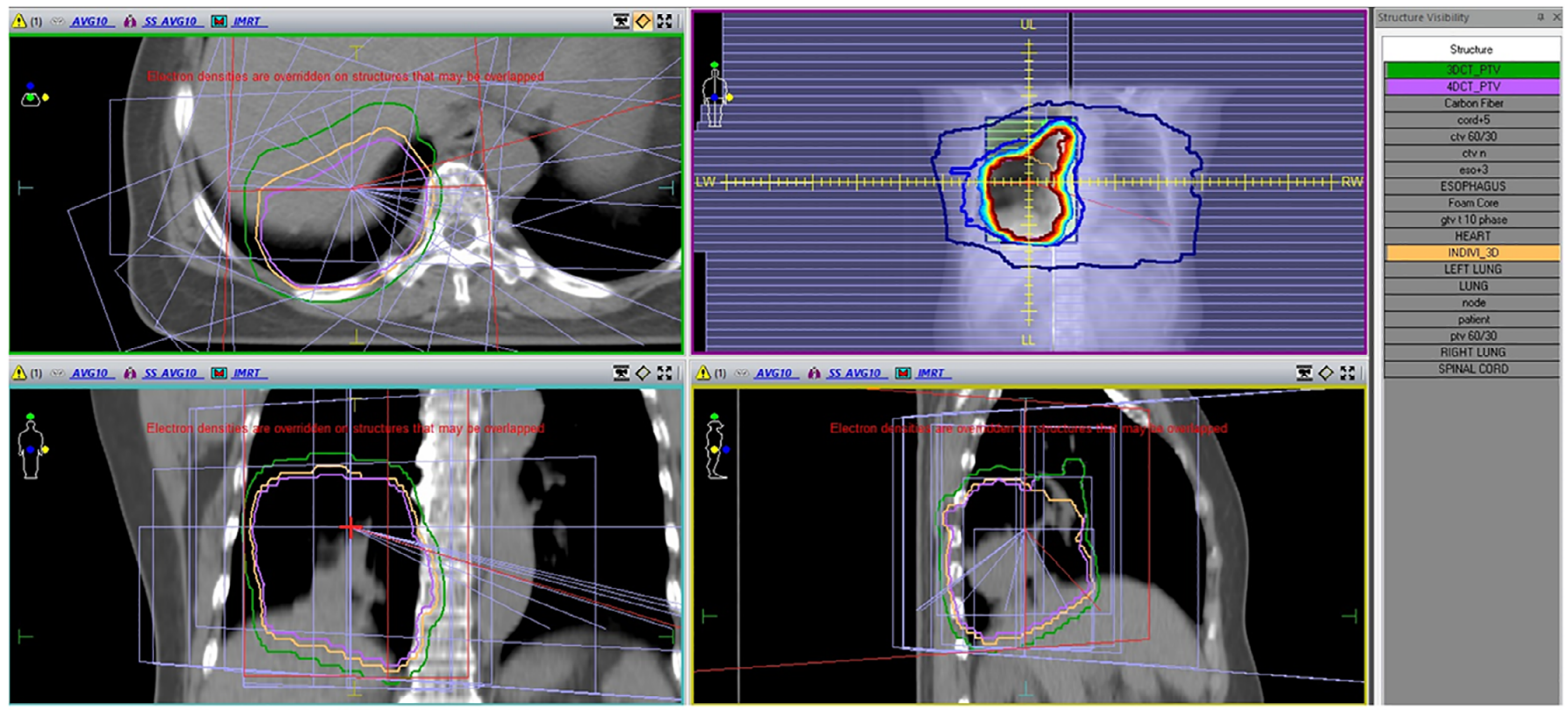
Representative patient case comparing treatment plans generated using conventional 3DCT, individualized 3D (Indiv‐3D), and 4DCT‐based approaches.

A representative patient case (Figures 8, 9) demonstrates anisotropic tumor motion predominantly along the superior–inferior axis and its clinical translation, where the individualized 3D (Indiv‐3D) approach achieves reduced PTV with preserved target coverage and dose distributions comparable to the 4DCT‐based reference.

## 4 DISCUSSION

These findings are consistent with prior studies highlighting the importance of respiratory motion management in lung radiotherapy, while extending existing approaches through the integration of physics‐based modeling and clinical factor weighting. This study demonstrates that physics‐informed respiratory motion modeling can be translated into clinically actionable individualization of PTV margins for patients with locally advanced NSCLC. Beyond simple geometric margin reduction, the present work highlights several clinically relevant innovations that distinguish this approach from conventional isotropic margin strategies and from purely technical motion‐modeling studies.

First, this study does not introduce fundamentally new motion modeling or margin formulations. Instead, it integrates established PCA‐based motion analysis and the van Herk margin concept into a clinically applicable framework for individualized margin selection. Whereas previous PCA‐based approaches have emphasized motion accuracy metrics such as reconstruction error or correlation coefficients,^21^the present analysis deliberately anchors motion modeling outcomes to clinically interpretable endpoints, including PTV volume, lung V20, mean lung dose, and estimated NTCP. By linking motion modeling directly to toxicity‐related dosimetric parameters, the proposed approach addresses clinical decision‐making rather than technical performance alone.

Second, this work introduces a pragmatic translational pathway between standard 3DCT planning and full 4DCT‐based optimization. Although 4DCT is widely available, its routine use for margin optimization, dose accumulation, or adaptive planning may be constrained by staffing, time, or infrastructure. The primary advantage of the proposed framework lies in its ability to provide motion‐informed margin adaptation using standard 3DCT data, thereby reducing reliance on full 4DCT‐based planning workflows. In this setting, the PCA/SVD framework functions as an intermediate solution, enabling motion‐aware individualized margins without the need for respiratory gating, tumor tracking, or adaptive delivery. This approach may be particularly useful in clinical settings where 4DCT is available but not routinely utilized for motion‐integrated planning, or in high‐throughput environments where full 4D analysis is not feasible for every patient. This positioning is particularly relevant for centers seeking incremental clinical benefit without substantial infrastructural investment.^18^, ^19^, ^20^

Third, the incorporation of estimated NTCP as a central outcome metric represents an important clinical innovation. Reductions in lung V20 and mean lung dose were translated into corresponding decreases in estimated NTCP for radiation pneumonitis, providing a toxicity‐oriented interpretation of dosimetric improvement. Many dosimetric studies report DVH changes in isolation; in contrast, the present work contextualizes these changes within established dose–toxicity relationships,^9^, ^16^, ^17^thereby strengthening the clinical relevance of the findings.

Fourth, the study demonstrates that lung sparing achieved through individualized margins does not incur a trade‐off in other critical organs at risk. Importantly, no statistically significant increases were observed in cardiac dose, oesophageal dose metrics, or maximum spinal cord dose, and all values remained within accepted clinical tolerance limits.^12^, ^29^This finding directly addresses a common clinical concern that margin reduction in one region may inadvertently increase dose elsewhere.

Fifth, the results suggest that the clinical benefit of individualized margins is not uniform across all patients but is modulated by tumor motion characteristics and anatomical location. Patients with larger superior–inferior motion amplitudes, particularly those with lower‐lobe tumors, demonstrated greater reductions in PTV volume and lung dose. This observation supports a selective personalization paradigm, in which motion‐aware margin strategies may be most impactful in well‐defined patient subgroups rather than universally applied.

Although amplitude‐based binning was used in this study to ensure consistent motion representation, the proposed framework is not limited to a specific binning strategy. Phase‐based binning may also be used; however, differences in temporal sampling and sensitivity to breathing irregularities may influence the extracted motion patterns. Future work is needed to systematically evaluate the impact of binning strategy on model performance. The framework is adaptable to different binning strategies as long as temporally ordered respiratory phases are available.

### 4.1 Clinical implications and implementation considerations

The proposed framework should not be interpreted as a replacement for 4DCT‐based planning. Instead, it represents a 4DCT‐informed approach, where motion characteristics derived from 4DCT datasets can guide margin selection within conventional 3DCT workflows. This paradigm enables the use of motion‐informed margins without requiring full 4DCT‐based planning or advanced motion management for every patient. In clinical practice, such an approach may be particularly useful in high‐throughput centers where 4DCT is available but not routinely utilized for detailed motion analysis or adaptive planning.

As such, implementation can be achieved within existing planning systems and quality assurance frameworks. The computational requirements are modest, and margin derivation can be integrated into the treatment planning process without prolonging simulation or delivery times (Method S3, Figures S3.1).

Importantly, this approach may be particularly valuable in high‐throughput centers or in resource‐limited settings, where advanced motion‐management technologies are not routinely available. By providing a motion‐aware alternative to population‐based isotropic margins, the PCA/SVD framework offers a pathway toward personalized radiotherapy that balances clinical benefit with operational feasibility. In this sense, the method complements, rather than replaces, more sophisticated motion‐management strategies.

Several limitations should be acknowledged. This study was conducted on a single‐institution cohort of 61 patients, which may limit the generalizability of the findings. Although the sample size is comparable to previous studies in motion modeling, external validation in larger and multi‐institutional datasets is required.^23^, ^24^, ^25^

The proposed framework should therefore be considered as a proof‐of‐concept and a clinically motivated, hypothesis‐generating approach rather than a definitive margin standard. Despite the moderate sample size, the consistency of motion patterns and dosimetric trends observed across patients supports the robustness of the proposed approach. A further limitation of this study is that breathing irregularity and intra‐fraction variability were not explicitly modeled. While the proposed framework captures dominant motion patterns derived from 4DCT, it assumes relatively regular respiratory cycles and may not fully represent transient or irregular motion behavior.

This limitation is particularly relevant from a clinical perspective, as patients with irregular breathing may benefit most from individualized, motion‐informed margin strategies. The absence of explicit modeling for such variability may therefore limit the applicability and generalizability of the proposed approach in these patient populations. Future work should incorporate more advanced motion characterization techniques, such as irregular breathing simulation, time‐resolved motion analysis, or integration of respiratory signal variability, to improve robustness in these scenarios.

While the flowchart provides a quantitative framework for margin calculation, the final clinical decision integrated plan evaluation metrics, such as DVH and OAR constraints, with the Radiation Oncologist's professional judgment (Method S3).

### 4.2 Comparative analysis with previous studies

To contextualize the performance of the proposed PCA‐based motion modeling approach, a quantitative comparison was conducted against conventional 3DCT and 4DCT‐based planning strategies (Table 8). The results demonstrate that motion‐informed margin adaptation can reduce PTV volume while maintaining target coverage.

**TABLE 8:** Quantitative comparison between the proposed PCA‐based margin approach and previously reported motion management strategies.

Metric	Current study (PCA‐based)	Heard et al. (2019)^23^	Admiraal et al. (2008)^24^	Kawahara et al. (2025)^25^
Technique	Individualized 3DCT planning	4DCT phase‐based ITV	4DCT motion‐weighted ITV	Virtual 4D‐VMAT
PTV volume (cm^3^)	**460.2 → 410 (−11%)**	Not explicitly reported	Reduced (no numeric value)	Not explicitly reported
Lung V20 (%)	**40.5 → 30.0 (−10.5%)**	Not reported	∼5%–8% reduction	Not reported
Target coverage	Maintained (D95/V95)	Improved coverage	Maintained	Maintained
Clinical focus	NTCP, toxicity	Geometric robustness	Dosimetric accuracy	Dosimetric sparing
Implementation	Standard 3DCT ± model	Full 4DCT required	4D dose accumulation	Advanced 4D system

Specifically, the individualized margin approach (Indiv‐3D) reduced the mean PTV volume from 460.2 ± 179.0 cm^3^ (Iso‐3D)–410 ± 170 cm^3^, reflecting a more efficient representation of patient‐specific tumor motion. This finding is consistent with prior studies highlighting the limitations of population‐based isotropic margins and the benefits of motion‐aware strategies.^23^, ^24^Unlike conventional approaches, the proposed method incorporate directional motion characteristics, enabling anisotropic margin design that better aligns with the dominant superior–inferior respiratory motion.

The geometric reduction in PTV translated into meaningful dosimetric improvements. Lung V20 decreased from 40.5% ± 8.6–30.0% ± 5.2, and mean lung dose was reduced from 18.3 ± 4.0 Gy–17.2 ± 2.8 Gy, approaching established QUANTEC constraints. These findings are in agreement with previous reports emphasizing the benefit of motion‐adaptive strategies, particularly in tumors with substantial respiratory displacement.^22^, ^25^

Furthermore, the reduction in estimated NTCP (from approximately 18%–22% to10–14%, p ≪ 0.05; Figure 7) provides a clinically relevant interpretation of these dosimetric gains. Importantly, this improvement was achieved without increasing doses to other critical organs such as the heart, esophagus, and spinal cord, supporting the clinical feasibility of the proposed approach.

While geometric overlap between target volumes was not explicitly assessed, dosimetric endpoints and target coverage metrics were prioritized as clinically meaningful indicators. The proposed method is therefore best interpreted as a motion‐informed strategy that improves planning efficiency and dosimetric outcomes. However, these findings should be understood in the context of dosimetric surrogates rather than direct clinical outcome data.

### 4.3 Economic feasibility and clinical implementation priorities

The proposed PCA‐based framework offers a pragmatic approach that balances dosimetric precision with clinical feasibility. By integrating motion characterization into margin design, the method addresses common operational constraints in contemporary radiotherapy workflows.

From an implementation perspective, the approach can be incorporated into existing clinical practice with minimal additional burden. It relies on standard 4DCT datasets and offline analysis, avoiding the need for advanced motion management technologies such as respiratory gating or real‐time tumor tracking. This makes the method particularly suitable for high‐throughput centers or settings where full motion‐integrated 4D planning is not routinely feasible.

Compared with conventional isotropic margin expansion, the individualized approach provides a more efficient representation of patient‐specific motion. As shown in Table 8, this results in reduced PTV volumes and corresponding improvements in lung dose metrics, without compromising target coverage or increasing dose to other critical organs.

Importantly, the clinical benefit of this strategy is not uniform across all patients. The greatest gains were observed in tumors located in the middle–lower lobes and in cases with larger superior–inferior motion amplitudes. These findings support a selective implementation strategy, where individualized margins are prioritized for patients most likely to benefit.

As illustrated in Figure 1, the proposed workflow serves as an intermediate solution between conventional 3DCT‐based planning and fully motion‐integrated 4D approaches. Rather than replacing 4DCT, it leverages prior motion information to enable motion‐informed margin selection within standard 3DCT workflows.

Overall, the framework provides a clinically feasible and resource‐efficient strategy for incorporating motion information into treatment planning, with favorable dosimetric outcomes and preserved target coverage.

## 5 CONCLUSION

Physics informed PCA/SVD respiratory motion modeling enables clinically meaningful individualization of PTV margins in locally advanced NSCLC. This pragmatic approach improves lung dosimetry and estimated toxicity risk while preserving routine clinical workflows, supporting its role as a translational motion aware planning strategy in contemporary lung radiotherapy.

## AUTHOR CONTRIBUTIONS

Dang.Q.H. conceptualized the study, performed data analysis, developed methodology, and drafted the manuscript. All authors reviewed and approved the final manuscript.

## CONFLICT OF INTEREST STATEMENT

The authors declare no conflicts of interest.

## ETHICS STATEMENT

This retrospective study was approved by the Institutional Review Board of Military Hospital 175 (Approval No. ĐT2021.24/BV175) and conducted in accordance with the Declaration of Helsinki. The requirement for informed consent was waived owing to the retrospective study design.

## Supporting information



Supporting Information

## Data Availability

The data supporting the findings of this study are available from the corresponding author upon reasonable request.
