# Supplementary material for: PCA‐based respiratory motion modeling for individualized PTV margin optimization in NSCLC radiotherapy
Source: J Appl Clin Med Phys. 2026 Jun 7;27(6):e70652. doi: 10.1002/acm2.70652 (PMC13243196; doi:10.1002/acm2.70652)
Supplement: Supplementary file 1 — Supporting Information [file ACM2-27-e70652-s001.docx]

**S1. Accuracy and Agreement Analysis Using RMSE, R², and Bland–Altman Methods.**

To comprehensively evaluate the accuracy and agreement of the proposed PCA–sinusoidal motion model, root mean square error (RMSE), coefficient of determination (R²), and Bland–Altman (BA) analyses were performed by comparing model-predicted motion trajectories with the reference motion derived from 4DCT. RMSE was used to quantify the magnitude of residual errors, while R² assessed the goodness-of-fit and the proportion of variance in the observed motion explained by the model. Bland–Altman analysis was further applied to evaluate systematic bias and limits of agreement between the two methods across the superior–inferior (S–I), anterior–posterior (A–P), and left–right (L–R) directions. Together, these complementary metrics provide a robust and clinically meaningful assessment of both precision and reliability of the proposed motion modelling approach.

**
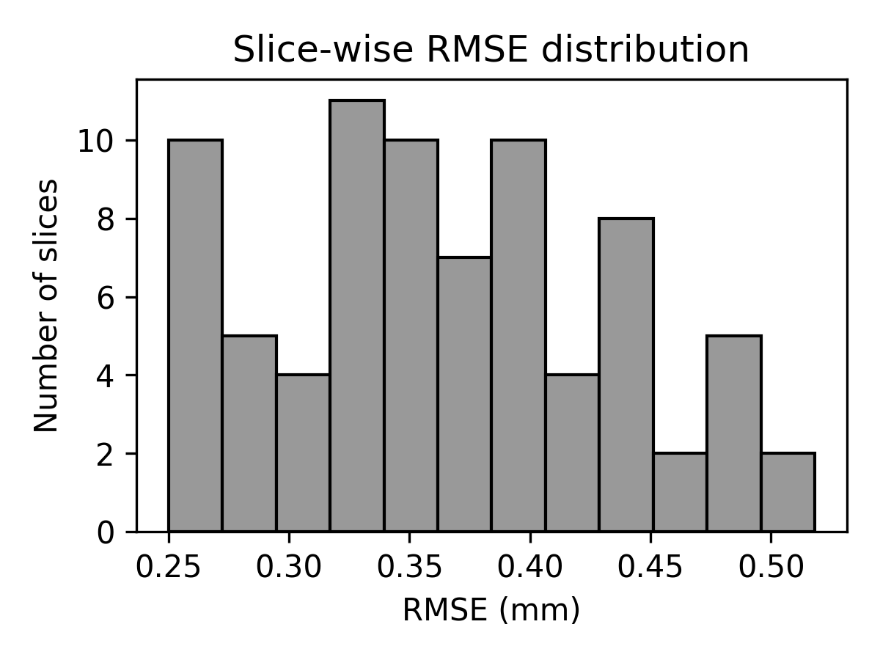
**

**Figure S1.1**. Distribution of slice-wise root-mean-square error (RMSE) for the PCA–sinusoidal respiratory motion model across all analysed axial slices (n = 78). The histogram demonstrates submillimetric modelling accuracy for the majority of slices.

**
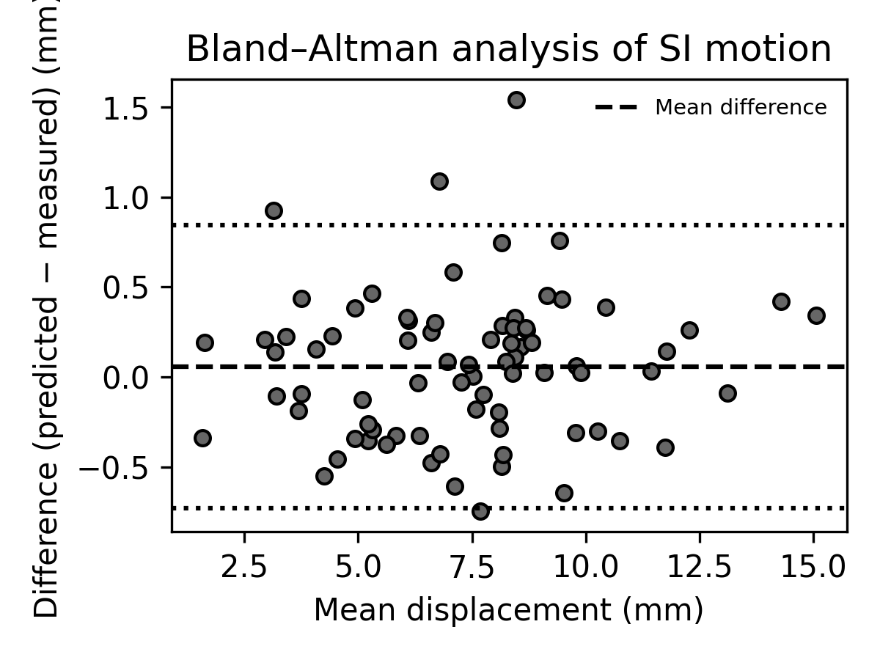
**

**Figure S1.2**. Bland–Altman analysis comparing model-predicted and measured superior–inferior tumour displacement. Solid line indicates mean difference; dotted lines represent the 95% limits of agreement

**S2. Sinusoidal Model Based on PCA**

The sinusoidal model was constructed from principal component analysis (PCA) of deformation vector fields derived via B-spline deformable registration.

The first three principal components (PC1–PC3) captured the dominant respiratory motion, with cumulative explained variance exceeding 90%. As shown in Figure S3.1, PC1, PC2, and PC3 accounted for approximately 72%, 15%, and 8% of the variance, respectively, yielding a cumulative variance of 95%. This supports the use of a reduced-order model for respiratory motion representation. Following Gong et al. (2024) [17]. Model validation demonstrated strong agreement between predicted and observed motion (R² = 0.78–0.86; RMSE = 0.3–0.5 mm), confirming the robustness of the sinusoidal representation.

**
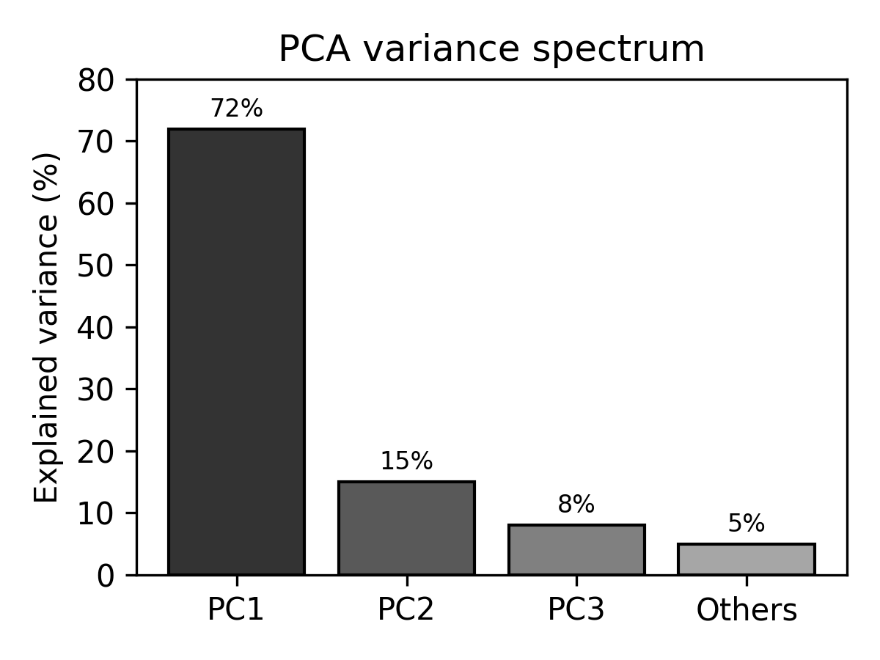
**

**Figure S2.1.** Explained variance spectrum of principal components derived from deformation vector fields. The first three components (PC1–PC3) account for approximately 95% of the total variance, indicating that respiratory motion can be effectively represented using a low-dimensional model.

**Supplementary Table S1.** Comparison of OAR dosimetric parameters among conventional 3DCT, individualized-3D, and 4DCT planning strategies. P-values represent overall comparisons across planning strategies using repeated-measures ANOVA

| OAR | Metric | 3DCT | Indiv-3D | 4DCT | p-value |
| --- | --- | --- | --- | --- | --- |
| Heart | Dmean (Gy) | 12.1 ± 4.5 | 11.8 ± 4.2 | 11.6 ± 4.3 | 0.42 |
| Esophagus | Dmean (Gy) | 18.3 ± 5.2 | 17.9 ± 5.0 | 17.6 ± 5.1 | 0.37 |
| Spinal cord | Dmax (Gy) | 43.2 ± 2.1 | 42.8 ± 2.0 | 42.5 ± 2.2 | 0.31 |

------------------------------------------------------------------------------------------

**Supplementary Methods S3**. Clinical Decision-Making Framework for Technique Selection

**S3.1. Overview**

This supplementary section describes a clinical decision-making framework developed to support the selection of simulation and treatment planning techniques in lung radiotherapy. The framework integrates physics-based motion modeling with clinically relevant factors to guide the choice among conventional three-dimensional computed tomography (3DCT), individualized 3DCT (PCA–sinusoidal model), and four-dimensional computed tomography (4DCT).

The proposed approach is intended as a **decision-support tool** to optimize technique selection while maintaining clinical flexibility.

**S3.2. Initial Imaging and Clinical Assessment**

All patients undergo standard **3DCT simulation** as the initial imaging modality. During this stage, the following clinical factors are systematically assessed:

- Tumor location (upper, middle, or lower lobe)
- Tumor stage (T1–T4) and size
- Respiratory pattern (regular vs. irregular breathing)
- Tumor proximity to organs at risk (OARs)
- Treatment intent (curative vs. palliative)
- Institutional resources (availability of 4DCT, CBCT, motion management tools)

These factors provide essential context for interpreting motion-related uncertainties.

**S3.3. Directional Motion Margin Estimation**

Respiratory motion is incorporated into margin design using a directional margin model:

$$M_{d}=\sqrt{\left( 2.5\Sigma)^{2}+(0.7\sigma)^{2}+(\alpha\cdot F_{pos}\cdot F_{T}\cdot A_{mean,d})^{2} \right.}$$

where:

- $M_{d}$: margin in direction $d$(S–I, A–P, L–R)
- $\Sigma$: systematic setup error
- $\sigma$: random setup error
- $A_{mean,d}$: mean motion amplitude in direction $d$
- $F_{pos}$: tumor location factor
- $F_{T}$: tumor stage factor
- $\alpha$: scaling factor for motion contribution

The superior–inferior direction ($M_{SI}$) is typically the dominant component and is used for motion risk stratification.

**S3.4. Motion Risk Stratification**

Patients are categorized into three motion risk groups based on $M_{SI}$:

| Risk Group | Suggested Criteria | Clinical Interpretation | Recommended Technique |
| --- | --- | --- | --- |
| Low Risk | (M_SI_ <3.5) mm; typically upper-lobe tumors and T3–T4 stage | Limited respiratory motion; geometric uncertainty is low | Conventional **3DCT** |
| Intermediate Risk | (M_SI_ = 3.5–4.5) mm; e.g., upper-lobe T1–T2 or lower-lobe T3–T4 | Moderate motion; requires individualized margin adaptation | **Individualized-3DCT (PCA–sinusoidal)** |
| High Risk | (M_SI_ > 4.5) mm or middle/lower-lobe tumors at T1–T2 stage | Large respiratory motion; high geometric uncertainty | **4DCT (ITV-based planning)** |

These thresholds are derived from observed motion patterns and serve as general guidance.

**S3.5. Technique Selection Strategy**

Based on motion risk classification, the following strategy is proposed:

- **Low-risk patients:**
  Conventional **3DCT** is typically sufficient, as motion-related uncertainties are limited.
- **Intermediate-risk patients:**
  **Individualized-3DCT (PCA–sinusoidal model)** is recommended to enable patient-specific margin adaptation and reduce unnecessary PTV expansion.
- **High-risk patients:**
  **4DCT** is strongly recommended to accurately capture respiratory motion and define the internal target volume (ITV).

**S3.6. Target Volume Construction**

The planning target volume (PTV) is generated according to the selected technique:

- **3DCT:** isotropic margin expansion based on institutional protocols
- **Individualized-3DCT:** directional margins using $M_{SI},M_{AP},M_{LR}$
- **4DCT:** ITV derived from respiratory phases, followed by setup margin expansion

**S3.7. Plan Evaluation Criteria**

Treatment plans are evaluated using standard dosimetric metrics:

- Target coverage (e.g., D95)
- Dose–volume histogram (DVH) parameters
- Lung dose metrics (V20, mean lung dose)
- OAR dose constraints
- Conformity index (CI) and homogeneity index (HI), when applicable

**S3.8. Role of Clinical Judgment**

It is important to emphasize that the proposed framework provides general recommendations only.

**S3.9. Summary**

Although the proposed framework provides structured guidance for motion-risk stratification and technique selection, the final treatment planning decision should remain individualized and clinically supervised.

In routine clinical practice, the recommended strategy (conventional 3DCT, individualized-3DCT, or full 4DCT planning) should be interpreted together with additional patient-specific considerations, including:

• overall performance status
• respiratory reproducibility
• tumor proximity to critical organs
• image quality and registration reliability
• institutional workflow constraints
• physician experience and multidisciplinary consensus

Therefore, the framework is intended to function as a decision-support tool rather than a rigid treatment selection algorithm. Final plan approval should integrate dosimetric evaluation, motion assessment, and clinical judgment by the radiation oncology team.


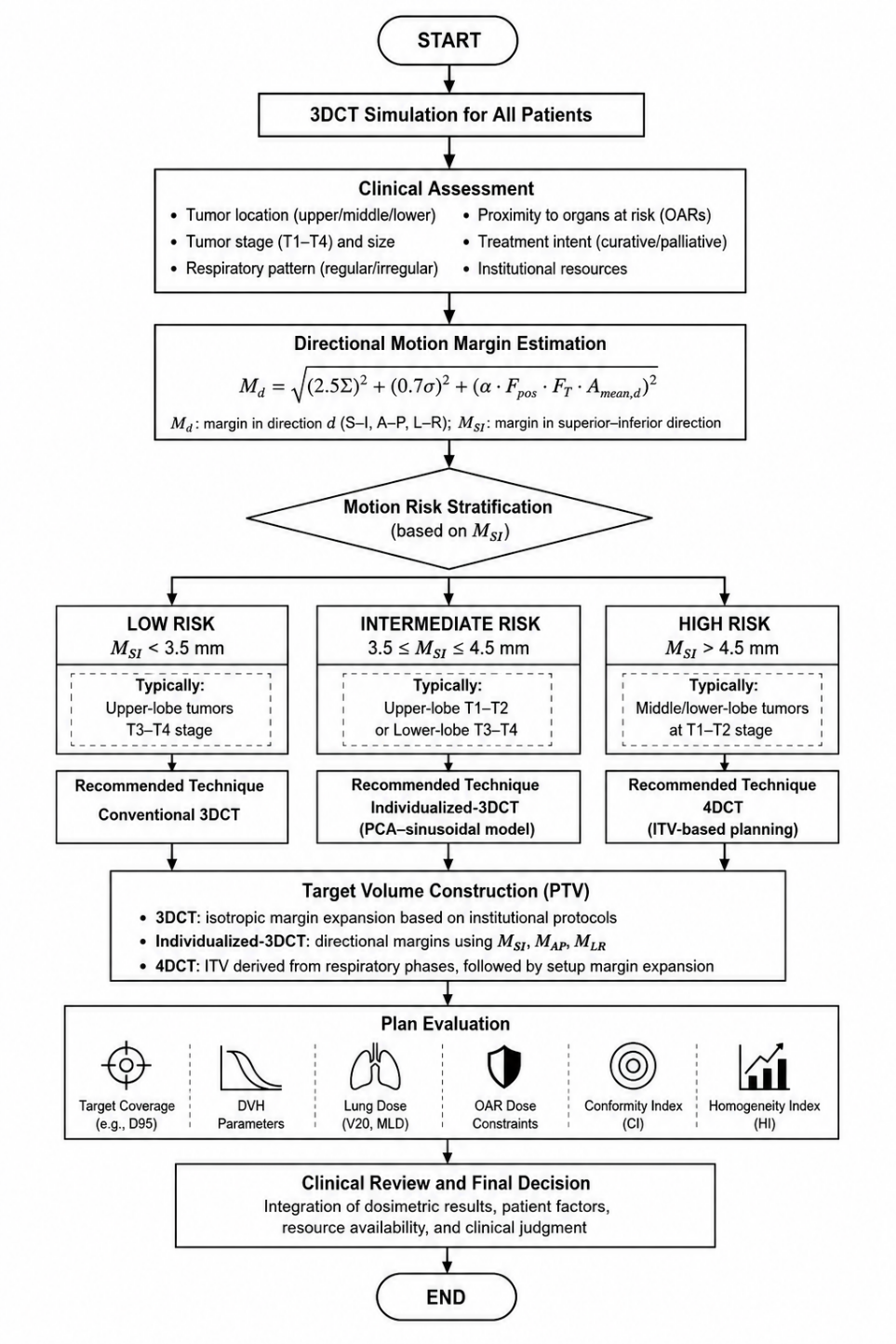


**Figure S3.1**. A step-by-step procedural map from initial clinical assessment and directional margin calculation to motion risk stratification and the final treatment plan evaluation for lung tumor radiotherapy. Parameters and thresholds are intended for stage IIIA–IIIC NSCLC patients and require external validation before application to other disease stages or treatment settings.
